# Experimental Researches into a New Excretory Function of the Liver; Consisting in the Removal of Cholesterine from the Blood, and Its Discharge from the Body in the Form of Stercorine

**Published:** 1862-11

**Authors:** Austin Flint

**Affiliations:** Professor of Physiology and Microscopy in the Bellevue Medical College, New York; &c.


### EXPERIMENTAL RESEARCHES INTO A NEW EXCRETORY FUNCTION OF THE LIVER, &c.

Bx AUSTIN FLINT, Jr., M.D., Professor of Physiology and Microscopy in the Bellevue Medical College, New York; &c.

(*Continued from page* 630.)

*Functions of the cholesterine.*—By experiments which I have performed upon the lower animals, and by certain facts which have been developed by observations on the human blood in health and disease, I conceive that I have been enabled to solve the problem of the function of cholesterine.

*Cholesterine is an excrementitious product, formed in great part by the destructive assimilation of the brain and nerves, separated from the blood by the liver, poured into the upper part of the small intestine with the bile, transformed in its passage down the alimentary canal into stercorine* (the seroline of Boudet, a substance differing very little from cholesterine), *and, as stercorine, discharged by the rectum.*

The quotation with which I prefaced this paper expresses the actual state of the science with regard to cholesterine. Still, though our actual knowledge of its function has been so slight, a few writers on chemical physiology and on physiology, taking the limited data on this subject, make reference to it as an effete substance. With regard to its relation to the brain, some think that it is formed in the brain and taken up by the blood, while others think that it is formed in the blood and deposited in the brain. All the views with regard to its effete properties are, of course, based on the supposition that it is discharged in the feces. Effete matters are discharged from the body, and this would find its exit by the anus, since it has never been detected in the urine. These conjectures have attracted little attention in the scientific world; and these views being based on the supposition that this substance is formed in the fecal matters, fall to the ground from the fact that no one as yet detected it in the feces. The fact that cholesterine is so generally considered an ingredient of the feces may be thus explained. It is poured into the alimentary canal with the bile; no one has shown what becomes of it, the chemistry of the feces being little understood, and therefore it has been assumed that it is found in the feces. That the facts which we have with regard to cholesterine render its effete properties possible, and, perhaps, probable, is certainly true; but these facts are merely sufficient to enable the scientific investigator to address an intelligent inquiry to nature on this subject; they do not resolve the question. In the experiments which form the basis of this article, the inquiry was made and the answer obtained; some others have, without much reflection, apparently, made simple statements which approximate in some degree to the facts. The only way these assertions could be sustained is by the labor which I have expended in eliciting from nature a reply to my interrogatories.

The works which I have had an opportunity of consulting, where any decided opinion relative to the function of cholesterine has been expressed, are those of Carpenter, Lehmann, Mailhe, and Dalton.*

Carpenter, in the fifth American edition of his Human Physiology, 1853, has the following with regard to the function of cholesterine.

"It is also stated to be a constituent of the nervous tissue, having been extracted from the brain by Couerbe, and other experimenters; but it may be doubted whether this is not rather a product of the disentegration of nerve-substance, which is destined to be taken back into the blood for elimination by the excretory apparatus, like the kreatine which may be extracted from the juice of flesh, or the urea which is obtainable from the vitreous humor of the eye, both being undoubtedly excrementitious matters. For cholesterine is a characteristic component of the biliary excretion, and is closely related to its peculiar acids; so that it can scarcely be looked upon in any other light than as an excrementitious product, the highest function of which is to assist in the support of the calorifying process. It is frequently separated from the blood as a morbid product; thus it is often present in considerable quantity in dropsical fluids, and particularly in the contents of cysts; and it may be deposited in the solid form in degenerated structures, tubercular concretions, &c."†

In Lehmann, we find the following on this subject:—

"Judging from the mode of its occurrence, we must regard it as a product of decomposition; but from what substances and by what process it is formed, it is impossible even to guess. Notwithstanding the similarity which many of its physical properties present to those of the fats, we can hardly suppose that it takes its origin from them, since the fats, for the most part, become oxidized in the animal body, whereas in order to form cholesterine, they must undergo a process of deoxidation."‡

I translate the following from the excellent work of Mialhe, on Chemistry applied to Physiology and Therapeutics, Paris, 1856, the paragraph entitled "Source of Cholesterine in the Animal Economy."

* These authors are quoted in the order in which their publications appeared.

† Carpenter's Principles of Human Physiology, page 74. Philadelphia, 1853.

‡ Physiological Chemistry, by Professor C. G. Lehmann, vol. i. 248. Philadelphia, 1855.

"We have just examined in what manner the fatty bodies penetrate into the blood. Some eminent *savans* have held that the fatty matters from the exterior are the only ones which exist in the economy, and that it is incapable of producing these in itself. Now it is an opposite opinion which tends to predominate, and the majority of physiologists think that certain fatty bodies take origin in the very substance of our organism. This last mode of origin seems at least incontestible for the cholesterine, which has not yet been found in the vegetable kingdom.

"But what are the chemico-physiological reactions which preside over the development of this particular fatty substance?

"There are for us two modes for comprehending the formation of the cholesterine at the expense of the elements of the blood. Cholesterine may come from the fatty matters; it would be, in this case, like the final result or last stage of chemical modifications which the fatty matters undergo in the animal economy,

"This manner of viewing it is slightly probable; for, in order that it should be true, it would be necessary that the fatty bodies, in oxidizing, should give rise to a compound richer than they in carbon. We know, indeed, that cholesterine is, of all fatty bodies, the one which contains the most carbon.

"We think that we should reject that opinion and stop at the following:

"The production of cholesterine may be attributed to a transformation of the albuminoid materials, a transformation analogous to that which has been pointed out by M. Blondeau de Carrolles in cheese, and which that chemist has designated under the name of adipose fermentation. The large proportion of carbon which the cholesterine contains, and which approximates it to albuminous matters, would come to the support of that point of view. The retardation of the circulation, and the deficiency of oxidation which is the consequence of it, explains also why the cholesterine is in much greater proportion in the closed cavities than in the blood itself.

"Whichever it may be of these two opinions, it is incontestible for us that, if the cholesterine be not burned with the other matters proper to respiratory alimentation, it is solely on account of its chemical inertia; cholesterine, indeed, is to fatty matters what mannite is to saccharine substances—what urea is to albuminoid matters; that is to say, that it constitutes a kind of *caput mortuum*, of which the organism has only to free itself. It is certain, also, for us, that if the cholesterine is not found in all the excrementitious liquids, where most of the other products existing in the blood are found, it is solely on account of its insolubility.

"The preceding remarks explain perfectly, to our eyes at least, why the presence of cholesterine has never been established in the urine of man, either in the form of crystals, or 'calculi,' while this substance is found in the bile, where it very often forms calculi of considerable size. Cholesterine, indeed, is insoluble in acid liquids, such as the urine; while it is soluble in soapy liquids, such as the bile. Such is solely the reason why the cholesterine is excreted by the biliary passages."*

Finally in Dalton's *Treatise on Human Physiology*, we find the following paragraph in which the subject of cholesterine is considered:—

"CHOLESTERINE ($C_{25}H_{22}O$).—This is a crystallizable substance which resembles the fats in many respects, since it is destitute of nitrogen, readily inflammable, soluable in alcohol and ether, and entirely insoluable in water. It is not saponifiable, however, by contact with the alkalies, and is distinguished

* Chimie appliquee a la Physiologie et a la Therapeutique. Par M. le Docteur Mialhe. Page 191. Paris, 1856.

on this account from the ordinary fatty substances. It occurs, in a crystalline form, mixed with coloring matter, as an abundant ingredient in most biliary calculi, and is found also in different regions of the body, forming a part of various morbid deposits. We have met with it in the fluid of hydrocele, and in the interior of many encysted tumors. The crystals of cholesterine have the form of very thin, colorless transparent, rhomboidal plates, portions of which are often cut out by lines of cleavage parallel to the sides of the crystal. They frequently occur deposited in layers, in which the outlines of the subjacent crystals show very distinctly through the substance of those which are placed above. Cholesterine is not formed in the liver, but originates in the substance of the brain and nervous tissue, from which it may be extracted in large quantity by the action of alcohol. From these tissues it is absorbed by the blood, then conveyed to the liver, and discharged with the bile."*

The above extracts embrace all that I have been able to find bearing on the question of the function of cholesterine. The extracts from Mialhe and Dalton contain all that is said by them on this subject. Those from Carpenter and Lehmann contain only what bears on the function of this substance, the chemical details being omitted. Of the authors cited, Mialhe is the most extended on the subject, and is almost the only one who adduces any arguments to support his views; but his opinions are biased by the purely chemical view which he takes of the subject, and are involved with the ideas with reference to plastic and calorific food, now rejected by many eminent physiologists, and which, I conceive, will be so little supported by future advances in science, that they will soon be universally discarded, in the exclusive sense in which they are received by him. Putting these hypotheses aside, we examine the actual state of our science, with regard to cholesterine, and we find that the function, up to this time, has not been established. We will now proceed to the facts which tend to support the statement I have made on this point.

Cholesterine exists in the blood, from which it may be extracted in a state of purity, and estimated by the process which I have already indicated. Becquerel and Rodier have made analyses of the healthy human blood for this substance with the following results:—

| | |
|---|---|
| Venous blood of the male. . . . . | 0.09 pts. per 1,000 |
| " " " female, . . . | 0.09 " " " |

I have made a quantitative analysis of three specimens of healthy human blood with the following results:—

* A Treatise on Human Physiology, designed for the use of students and Practitioners of Medicine. By John C. Dalton, Jr., M.D., Professor of Physiology and Microscopic Anatomy in the College of Physicians and Surgeons, New York, &c. Page 189. Philadelphia, 1861.

| | Quantity of Blood. grains. | Cholesterine. grains. | Proportion per 1,000 pts. |
|---|---|---|---|
| Venous blood from the arm; male æt. 35 | 312.083 | 0.139 | 0.445 |
| Do. do. do. (colored) æt. 22 | 187.843 | 0.123 | 0.658 |
| Do. do. do. æt. 24 | 102.680 | 0.077 | 0.751 |

These three analyses were all carried on at the same time, and each specimen subjected to precisely the same process. The results show a wide range within the limits of health. The difference was not due to any variation relating to the digestive process, as the specimens were all drawn at the same time, and were taken from prisoners on Blackwell's Island, who were subjected to the same diet, and ate at the same time. It will be seen by this table that I have obtained from five to eight times as much as is indicated by Becquerel and Rodier. I can only explain this by the fact that I operated on the whole blood, while they only analyzed the serum. Boudet states that it is necessary to make three to four copious bleedings, and mix the serum in order to obtain a sufficient quantity for a satisfactory analysis. I have operated on about fifty grains of blood with success, and have no doubt but that I would be able to extract the cholesterine in a crystalline form, and estimate its quantity in fifteen and twenty grains. The purity of the extract can easily be demonstrated by a microscopic examination. I conclude, then, that a much larger quantity of cholesterine exists normally in the blood than has been supposed, and that its variations, in different persons, within the limits of health, are considerable.

The next question which naturally arises is the origin of the cholesterine. When we examine the situations in which it is found, we find that it exists in largest quantity in the substance of the brain and nerves. It is also found in the substance of the liver, probably in the bile which is contained in this organ, and the crystalline lens, but with these exceptions it is found only in the nervous system and blood. Two views present themselves with regard to its origin. Cholesterine is deposited in the nervous matter from the blood, or is formed in the brain and taken up by the blood. This is a question, however, which can be settled experimentally, by analyzing the blood for cholesterine as it goes to the brain by the carotid, and as it comes from the brain by the internal jugular. The cholesterine being found also in the nerves, and, of course, a large quantity of nervous matter existing in the extremities, it is desirable at the same time to make an analysis of the venous blood from the general system.

With reference to this question the following experiment was made:—

*Exp.* 3. A medium sized dog, about six months old, fasting, was put under the influence of ether. The carotid and internal jugular were exposed on the left side, and the animal allowed to come out from the effects of the anæsthetic. Two hours after, he was again etherized, and the blood taken from the following vessels in the order in which they are named: 1, Internal jugular; 2, Carotid; 3, Vena cava; 4, Hepatic veins; 5, Hepatic artery; 6, Portal vein. In the operation of drawing the blood from the abdominal vessels, immediately after opening the abdomen a ligature was applied to the vena cava and a little blood taken, which prevented the blood from the inferior extremities from mixing with the hepatic blood. The blood was then taken from the hepatic veins, a matter of some difficulty, as it is always more or less mingled with blood returning through the thoracic vena cava, and a ligature applied to the hepatic artery and portal vein. The blood was then drawn from the hepatic artery and portal vein.* A quantity of bile was then taken from the gall-bladder, and a portion taken from the substance of the brain. These specimens were received into carefully weighed vessels and weighed; but as I failed to make a quantitative analysis, my process of extraction not having been perfected, it is unnecessary to enter into details. They were then dried and pulverized, treated with ether, evaporated, the residue extracted with hot alcohol, allowed to evaporate spontaneously, and examined with magnifying powers of 70, 270, and 400 diameters successively. The residue of the bile and brain were found to consist of nearly pure cholesterine, but in all the other specimens, excepting that from the internal jugular, the appearance of cholesterine was doubtful. They all contained, with masses of

* The operation of collecting the blood from any particular vessel is by no means so easy as might at first be supposed. The greatest care is necessary in order to obtain it unmixed. This is particularly so in the case of the hepatic vein, the unmixed blood from which it is exceedingly difficult to obtain. In drawing blood, the operation must be done as rapidly as possible to avoid the derangements of the circulation which arise from exposure of the vessels, pressure, etc. In taking blood going to and coming from a part, it must always be taken from the vein first; as ligating or compressing the artery would of course arrest the circulation. As the blood in the arterial system is not subject to the same changes in composition as the blood in the different veins, any specimen of the arterial blood will represent the blood going to a part, unless, like the liver, it receives blood from the venous system. The collection of blood I have found the most difficult part of these investigations.

ordinary fat, crystals of stercorine.* There were a few distinct plates of cholesterine in the specimen from the internal jugular. The specimens were then treated with a solution of caustic potash and set aside. In two days, part of the potash was removed with bibulous paper and portions of the precipitates taken out, placed upon slides, and examined microscopically with one-sixth and one-twelfth inch objectives successively. The watch-glasses were then set aside, carefully protected from the dust, and examined again ten days after, when they had become entirely dry. The following was the result of the examinations of the extracts of blood from the carotid, internal jugular, vena cava, and the extract of the brain. The examination of the other specimens has nothing to do with the question now under consideration, and their description is deferred.

*Blood from the carotid artery.*—First examination, three days after the operation, discovered a large number of small crystals of stercorine and masses of fat; but after the most careful examination, prolonged for two hours, I failed to discover any crystals of cholesterine. The appearance is represented in Fig. 2.

The second examination, eleven days after, discovered a small quantity of cholesterine mixed with the matters noted in the first examination. This appearance is represented in Fig. 3.

*Substance of the brain.*—All the microscopic examinations of the extract from the brain showed crystals of cholesterine in large quantity. The crystals from the brain are described by Robin as being thinner and more elongated than those found in other situations.† This peculiarity I also noticed. The appearance is represented in Fig. 4.

*Blood from the internal jugular.*—In the first examination of the specimen from the internal jugular, after the blood had been treated with ether, the ether allowed to evaporate, and the residue extracted with hot alcohol, well-marked plates of cholesterine were noted. At this time it could not be discovered in any of the other specimens of blood after the most careful and patient examination. After the caustic potash has been added, the

* Stercorine, or seroline, is a non-saponifiable fatty substance resembling the cholesterine in many of its chemical properties, but fusing at a much lower temperature. It was discovered in the serum of the blood by Boudet about 1833. It crystallizes in the form of needles, which will be more particularly described when we treat of the extraction of this substance from the feces. As I have found it in great abundance in the feces, and am disposed to doubt its existence as a natural constituent of the serum of the blood, I have called it *stercorine*, for reasons which will be more fully explained further on.

† Traite de Chimie Anatomique, Robin and Verdeil, tome iii. p. 57.

cholesterine was demonstrated in large quantity, with a few crystals of stercorine. The appearance is represented in Fig. 5, which was drawn eleven days after the blood was collected. Another examination was made on the following day, which showed, in addition to the cholesterine, a considerable quantity of stercorine. (See Fig. 6.)

*Blood from the vena cava.*—The extract of the blood from the vena cava, examined eleven days after the blood was drawn, showed a large quantity of stercorine and a few crystals of cholesterine. The cholesterine was distinct but not very abundant. (See Fig. 7.)

These experiments, the first that I made on this subject, demonstrate the following facts: 1. That the brain contains a large quantity of cholesterine (which had, however, been previously established). 2. That the blood going to the brain contains a small quantity of cholesterine, while the blood coming from the brain contains a large quantity. 3. That the blood coming from the lower extremities and pelvic organs contains more cholesterine than the blood carried to them by the arterial system.

It was only necessary to confirm these statements by further investigation, to be enabled to deduce from them the following important conclusion: *i. e.* That cholesterine is formed in some of the tissues of the body; and judging from the fact that the nervous tissue is the only one in which it is found, and that the blood gains it in its passage through the great nervous centre, it is formed, in great part, by the nervous system. After the first experiment, which almost confirmed the supposition with which I had started, I directed my attention to the perfection of a process by which I might make an accurate quantitative analysis of the blood for cholesterine, so as to be able to state positively that it gained cholesterine in its passage through certain organs, and furthermore to determine the amount of increase. After a number of experiments, I fixed upon the process which I have minutely described in the first part of this article, and made the following experiments for the purpose of ascertaining the quantity of cholesterine produced in the brain.

*Exp.* 4. A medium sized adult dog was put under the influence of ether and the carotid artery, internal jugular, and femoral veins exposed. Specimens of blood were drawn, first from the internal jugular, next from the carotid, and last, from the femoral vein. These specimens were received into carefully weighed vessels, and weighed.

They were then analyzed for cholesterine by the process described on pages 626–629, and the following results obtained:—

| | Quantity of Blood. grains. | Cholesterine. grains. | Cholesterine per 1,000 pts. |
|---|---|---|---|
| Carotid, . . . . | 179.462 | 0.139 | 0.774 |
| Internal jugular, . . | 134.780 | 0.108 | 0.801 |
| Femoral vein, . . . | 133.886 | 0.108 | 0.806 |

Percentage of increase in blood from the jugular, over the arterial blood, 3.488
Do. do. of blood from femoral vein, . . . . . 4.134

This experiment shows an increase in the quantity of cholesterine in the blood during its passage through the brain and an inerease, even a little greater, in the blood passing through the vessels of the posterior extremity. To facilitate the operation, however, the animal was brought completely under the influence of ether, which, from its action on the brain, would not improbably produce some temporary disturbance in the nutrition of that organ, and consequently interfere with the experiment. For the purpose of avoiding this difficulty I performed the following experiments without administering an anæsthetic.

*Exp.* 5. A small young dog was secured to the operating table, and the internal jugular and carotid exposed on the right side. Blood was taken, first from the jugular, and afterwards from the carotid. The femoral vein on the same side was then exposed and a specimen of blood taken from that vessel. The animal was very quiet under the operation, though no anæsthetic was used, so that the blood was drawn without any difficulty, and without the slightest admixture.

The three specimens were analyzed for cholesterine with the following results:—

| | Quantity of Blood. grains. | Cholesterine. grains. | Cholesterine per 1,000 pts. |
|---|---|---|---|
| Carotid, . . . . . | 143.625 | 0.679 | 0.967 |
| Internal jugular, . . | 29.956 | 0.046 | 1.545 |
| Femoral vein, . . . | 45.035 | 0.046 | 1.028 |

Percentage of increase in blood from the jugular, over arterial blood, 59.772
Do. do. of blood from the femoral vein, . . . . 6.308

*Exp.* 6. A large and powerful dog was secured to the operating table and the carotid and internal jugular exposed. Specimens of blood were taken from these vessels, first from the jugular, carefully weighed and analyzed for cholesterine in the usual way. The following results were obtained:—

| | Blood. grains. | Cholesterine. grains. | Proportion in 1,000 pts. |
|---|---|---|---|
| Carotid, . . . . . | 140.847 | 0.108 | 0.768 |
| Internal jugular, . . | 97.811 | 0.092 | 0.947 |

Percentage of increase in passing through the brain, . . . . 23.307

Exp. 5 shows a very considerable increase in the quantity of cholesterine in the blood passing through the brain, while it is comparatively slight in the blood of the femoral vein. The proportion of cholesterine is also large in the arterial blood compared with other observations.

Exp. 6 shows but a slight difference in the quantity of cholesterine in the arterial blood in the two animals; the proportion in the animal that was etherized being 0.774 pts. per 1,000, and in the animal that was not etherized 0.768 per 1,000, the difference being but 0.006; but, as I had suspected, the ether had an influence on the quantity of cholesterine absorbed by the blood in its passage through the brain. In the first instance the increase was but 3.488 per cent, while in the latter it was 23.307. Unfortunately the blood was not taken from the femoral vein. I intended to take blood from the abdominal organs, but after opening the abdomen the struggles of the animal were so violent that this was impossible, and he was killed.

What are our natural conclusions, from the preceding experiments, with regard to the origin of cholesterine in the economy? It has been found that the brain and nerves contain a large quantity of this substance, which is found in none other of the tissues of the body. The preceding experiments, especially Exps. 5 and 6, show that the blood which comes from the brain contains a much larger quantity of cholesterine than the blood which goes to this organ.

*The conclusion is, then, that it is produced in the brain, and thence absorbed by the blood.*

But the brain is not the only part where cholesterine is produced. It will be seen by Exp. 4 that there is 4.134 per cent, and in Exp. 5, 6.308 per cent of increase in the cholesterine in the passage of the blood through the inferior extremities, and probably about the same in other parts of the muscular system. In examining these tissues chemically, we find that the muscles contain no cholesterine, but that it is abundant in the nerves; and as we have found that the proportion of cholesterine is immensely increased in the passage of the blood through the great centre of the nervous system, taken, as the specimens examined were, from the internal jugular, which collects the blood from the brain and very little from the muscular system, it is rendered almost certain, that in the general venous system, the cholesterine which the blood contains is produced in the substance of the nerves.

If this be true, and if, as I hope to show, the cholesterine be a product of the destructive assimilation of nervous tissue, its production would be proportionate to the activity of the nutrition of the nerves; and anything which interfered to any great extent with their nutrition would diminish the quantity of cholesterine produced. In the production of urea by the general system, which is an analogous process, muscular activity increases the quantity, and inaction diminishes it, on account of the effect upon nutrition. In cases of paralysis we have a diminution of the nutritive forces in the parts affected, especially of the nervous system, which, after a time, becomes so disorganized, that although the cause of the paralysis be removed, the nerves cannot resume their functions. It is true we have this to a certain extent in the muscles; but it is by no means as marked as it is in the nerves. We should be able then to confirm the observations on animals, by examining the blood in cases of paralysis; when we should find a very marked difference in the quantity of cholesterine, between the venous blood coming from the paralyzed parts, and that from other parts of the body. With this in view I made analyses of the blood from both arms in three cases of hemiplegia, which seemed to me most suitable for such a comparison.

Case I. Sarah Rumsby, æt. 47, affected with hemiplegia of the left side. Two years ago she was taken with apoplexy, and was insensible for three days. When she recovered consciousness she found herself paralyzed on the left side. Said she had epilepsy four or five years before the attack of apoplexy. Now she has entire paralysis of motion on the affected side, with the exception of some slight power over the fingers, but sensation is perfect. The speech is not affected. The general health is good.

Case II. Anna Wilson, æt. 23, Irish, affected with hemiplegia of the right side. Four months ago she was taken with apoplexy, from which she recovered in one day with loss of motion and sensation on the right side. She is now improving and can use the right arm slightly. The leg is not so much improved, because she will make no effort to use it.

Case III. Honora Sullivan, Irish, æt. 40, affected with hemiplegia of right side. About six months ago she was taken with apoplexy, and recovered consciousness the next day, with paralysis. The leg was less affected than the arm, from the first. The cause was supposed by Dr. Flint, the attending physician, to be due to an embolus. Her condition is now about the same as regards the arm, but the leg has somewhat improved.

These cases all occurred at the Blackwell's Island Hospital. The treatment in all consisted of good diet, frictions, passive motion, and use of the paralyzed members as much as possible.

A small quantity of blood was drawn from both arms in these three cases. It was drawn from the paralyzed side, in each instance, with great difficulty, and but a small quantity could be obtained.

The specimens were all examined for cholesterine with the following results:—

*Table of Quantity of Cholesterine in Blood of Paralyzed and Sound Sides, in three cases of Hemiplegia.*

| | Blood. | Cholesterine. | Cholesterine per 1,000. |
|---|---|---|---|
| | grains. | grains. | |
| Case I. Paralyzed side. | 55.458 | — | The watch-glass contained 0.031 gr. of a substance, but the most careful examination failed to show a single crystal of cholesterine. |
| Do. Sound side. | 128.407 | 0.062 | 0.481. |
| Case II. Paralyzed side. | 18.381 | — | Same as Case I. |
| Do. Sound side. | 66.396 | 0.062 | 0.808. |
| Case III. Paralysed side. | 21.842 | — | Same as Case I. |
| Do. Sound side. | 52.261 | 0.031 | 0.579. |

The result of these examinations is very interesting: not a single crystal of cholesterine was found in any of the three specimens of blood from the paralyzed side, while about the normal quantity was found in the blood from the sound side. As the nutrition of other tissues is interfered with in paralysis, it is impossible to say positively from these observations alone, that the cholesterine is produced in the nervous system only. But the nutrition of the nerves is undoubtedly most affected; and this observation, taken in connection with the preceding experiments on animals, seem to settle where the cholesterine is produced.

We may extend our first conclusion, then, and state that the *cholesterine is produced in the substance of the nervous system.*

Before entering upon the character of cholesterine, and inquiring whether it be an excrementitious or a recrementitious product, we will endeavor to follow it out in the system and ascertain if there be any organ which separates it from the blood. In pursuing this question, the method will be adopted that has been employed in investigating its origin; that is, analyzing the blood as it goes to and comes from certain organs. The organ which we would be led first to examine is the liver, as it is the only gland, the product of which contains cholesterine, which, if not manufactured in the gland itself, must be separated from the blood.

In the first series of experiments which I performed on this subject, I endeavored to show on the same animal the origin of cholesterine in certain parts, and its removal from the body. In these experiments, which were only approximative, as I had not then succeeded in extracting the cholesterine perfectly pure, I commenced with the arterial blood, examining it as it went into the brain by the carotid, analyzing the substance of the brain, then analyzing the blood as it came out of the brain by the internal jugular, examining the blood as it went into the liver by the hepatic artery and portal vein, examining the secretion of the liver, then the blood as it came out of the liver by the hepatic vein, examining also the blood of the vena cava in the abdomen. The analyses of the blood from the carotid, internal jugular, and vena cava have already been referred to, page 678, in treating of the origin of the cholesterine. It will be remembered that there was a large quantity of this substance in the internal jugular, and but a small quantity in the carotid, showing that it was *formed* in the brain. I now give the conclusion of those observations, which bears upon the *separation* of the cholesterine from the blood.

*Exp.* 7. Specimens of blood were taken from the hepatic artery, portal vein and hepatic vein, and a small quantity of bile from the gall-bladder. These specimens were treated in the manner already indicated in Exp. 3; *i. e.*, evaporated and pulverised, extracted with ether, the ether evaporated, and the residue extracted with boiling alcohol, this evaporated, a solution of caustic potash added and then subjected to a microscopic examination.

*Blood from the portal vein.*—Microscopic examination of the extract from the portal vein showed quite a number of crystals of cholesterine, which are represented in Fig. 8. These were observed after the fluid had nearly evaporated.

*Blood from the hepatic artery.*—Microscopic examination of the extract from the hepatic artery, made after the fluid had nearly evaporated, showed a considerable amount of cholesterine; more than was observed in the preceding specimen. (See Fig. 9.) There were also observed a few crystals of stercorine, represented in Fig. 10.

*Blood from the hepatic vein.*—The first examination of the extract from the hepatic vein, which was made just before the potash was added, showed a number of fatty masses with some crystals of stercorine. The solution of potash was then added, and two days after, another careful examination was made, discovering nothing but fatty globules and granules. (See Fig. 11.) The watch-glass was then set aside, and was examined eleven days after, when the fluid had entirely evaporated. At this examination, a few crystals of cholesterine were observed for the first time. (See Fig. 12.) There were also a number of crystals of margaric and stearic acid.

*Bile.*—All the examinations of the extract from the bile showed cholesterine; the precipitate consisted, indeed, of this substance in a nearly pure state. Fig. 13 represents some of the crystals which were observed in this specimen.

This series of experiments being taken in connection with the first observations on the carotid and internal jugular, while the one series demonstrates pretty conclusively that cholesterine is formed in the brain, the other shows that it disappears, in a measure, from the blood in its passage through the liver, and is found in the bile. In other words, it is formed in the nervous tissue, and prevented from accumulation in the blood by its excretion by the liver. This suggests an interesting series of inquiries; and this fact, substantiated, would be as important to the pathologist as to the physiologist. But in order to settle this important question, it is necessary to do something more than make an approximative estimate of the quantity of cholesterine removed from the blood by the liver. The quantity which is thus removed in the passage of the blood through this organ should be estimated, if possible, as closely as the quantity which the blood gains in its passage through the brain. But this estimate is more difficult. The operation for obtaining the blood, in the first place, is much more serious than that for obtaining blood from the carotid and internal jugular. It is very difficult to obtain the unmixed blood from the hepatic vein; and the exposure of the liver, if prolonged, must interfere with its eliminative function, in the same way that exposure of the kidneys arrests, in a few moments, the flow from the ureter. It is probable, however, that the administration of ether does not interfere with the elimination of cholesterine by the liver as it does, apparently, with its formation in the brain. Anæsthetics, we know, have a peculiar and special action on the brain, but do not interfere with the functions of vegetative life, lik secretion and excretion; and, we would suppose, would not interfere with the depurative function of the liver. It is fortunate that this is the case, for the operation of taking blood from the abdominal vessels is immensely increased in difficulty by the struggles of an animal not under the influence of an anæsthetic, so much so, indeed, that I failed entirely in obtaining any blood from one animal (the one used in Exp. 6), which was not etherized. It was a very powerful dog, and his struggles were so violent that it was impossible to collect the blood accurately from the abdominal vessels, and the attempt was abandoned. With a view of settling the question of the disappearance of a portion of the cholesterine of the blood in its passage through the liver, by an accurate quantitative analysis, I repeated the operation for drawing blood from the vessels which go into, and emerge from the liver. In my first trial the blood was drawn so unsatisfactorily, and the operation was so prolonged, that I did not thing it worth while to complete the analysis, and abandoned the experiment. In the following one I was more successful.

*Exp.* 8. A good-sized bitch (pregnant) was brought completely under the influence of ether, the abdomen laid freely open, and blood drawn, first from the hepatic vein, and next from the portal vein. The taking of the blood was entirely satisfactory, the operation being done rapidly, and the blood collected without any admixture. A specimen of blood was then taken from the carotid to represent the blood from the hepatic artery.

The three specimens of blood were then examined in the usual way for cholesterine, with the following results:—

| | Blood. grains. | Cholesterine. grains. | Cholesterine in 1,000 pts. |
|---|---|---|---|
| Arterial blood, . . | 159.537 | 0.200 | 1.257 |
| Portal vein, . . . | 168.257 | 0.170 | 1.009 |
| Hepatic vein, . . . | 79.848 | 0.077 | 0.964 |

Percentage of loss in arterial blood in its passage through the liver, 23.309
Do. do. do. of portal vein, . . . . . 4.460

This experiment proves positively that there was good ground for supposing from Exp. 7, namely, that cholesterine is separated from the blood by the liver; and here we may note, in passing, a striking coincidence between the analysis in Exp. 6, when the blood was studied in its passage through the brain, and the one just mentioned, when the blood was studied in its passage through the liver. *The gain of the arterial blood in cholesterine in passing through the brain was* 23.307 *per cent, the loss of this substance in passing through the liver is* 23.309 *per cent.* There must be, of course, the same quantity separated by the liver that was formed by the nervous system, it being formed, indeed, only to be separated by this organ, its formation being continuous, and its removal necessarily the same, in order to prevent its accumulation in the circulating fluid. The almost exact coincidence between these two quantities, in specimens taken from different animals, though not at all necessary to prove the fact just mentioned, is still very striking.

It is shown by Exp. 8 that the portal blood, as it goes into the liver, contains but a small percentage of cholesterine over the blood of the hepatic vein, while the percentage in the arterial blood is large. The arterial blood is the mixed blood of the entire system, and as it probably passes through no organ before it gets to the liver which diminishes its cholesterine, contains a quantity of this substance, which must be removed. The portal blood, coming from a limited part the system, contains less of this substance, though it gives up a certain quantity. In the circulation of the liver, the portal system largely predominates, and is necessary to other important functions of this organ, such as the production of sugar and fat. Soon after the portal vein enters the liver, its blood becomes mixed with that from the hepatic artery,* and from this mixture the cholesterine is separated. It is only necessary that blood, containing a certain quantity of cholesterine, should come in contact with the bile-secreting cells, in order that this substance be separated. The fact that it is eliminated by the liver is proven with much less difficulty than that it is formed in the nervous system. In fact, its presence in the bile, the necessity of its constant removal from the blood, which is consequent on its constant formation and absorption by this fluid, are almost sufficient in themselves to warrant the conclusion that it is removed by the liver. This, however, is put beyond a doubt by the preceding analysis of the blood going to and coming from this organ.

Another link, then, is added to the chain of facts which make up the history of cholesterine. The first is that—

*Cholesterine is formed in the brain and nervous system, and absorbed by the blood.*

* According to Robin, the branches of the hepatic artery are distributed almost entirely in the interlobular plexuses, and on the walls of the hepatic duct and portal vein, and do not find their way into the substance of the lobules.—*Dictionnaire de Medecine, de Chirurgie, de Pharmacie, des Sciences accessoires et de l' Art veterinaire de* P. H. Nysten; *onzieme edition revue et corrige.* Par E. Littre et Ch. Robin. Paris, 1858. Article *Foie.*

The second, which has just been proven, is that—

*Cholesterine, formed in these situations, and absorbed by the blood, is separated from the blood in its passage through the liver.*

The next question, in following out this line of inquiry, is, What becomes of the cholesterine which is separated from the blood? This question is very easily answered, and necessitates only an examination of one of the products of the liver, the bile.

*The Bile.*—In the few remarks with which I have prefaced this article, I spoke of the various opinions which are held among physiologists with reference to the function of the bile —some regarding it as purely excrementitious, others placing it among the recrementitious fluids. I detailed experiments which led me to think that it had two distinct functions: one, which is recrementitious, and is probably concerned in digestion to an important degree, but which it is not designed to take up in this connection; the other, which is excrementitious, and which is necessarily taken up in our discussion of the important principle which we are now considering. A glance at the composition of the bile will show that it is an exceedingly complex fluid; and physiological investigations into the destination of certain of its ingredients, by Bidder and Schmidt, Dalton and others, have shown that they are not discharged from the body, but resorbed by the blood; though the failure to detect them in the portal blood by the appropriate tests, shows that in this resorption they probably undergo some alteration.* These substances, which have heretofore been considered the most important ingredients of the bile, though their function is obscure, are the glyco-cholate and tauro-cholate of soda, discovered by Strecker in the bile of the ox in 1848. The following is the composition of the bile given in Dalton's physiology, which is "based on the calculations of Berzelius, Frerichs, and Lehmann."†

* For a very complete account of the bile, with original investigations into the destination of the biliary salts, the reader is referred to an article published by Prof. John C. Dalton, Jr., in the *American Journal of the Medical Sciences*, October, 1857, and the chapter on bile in Dalton's Physiology.

† Dalton's Physiology, second edition, page 158.

*Composition of Ox Bile.*

| | |
|---|---|
| Water, | 888.00 |
| Glyco-cholate of soda, | 90.00 |
| Tauro-cholate of soda, | |
| Biliverdine, | 13.42 |
| Fats, | |
| Oleates, margarates, and stearates of soda and potassa, | |
| Cholesterine, | |
| Chloride of sodium, | 15.24 |
| Phosphate of soda, | |
| Phosphate of lime, | |
| Phosphate of magnesia, | |
| Carbonates of soda and potassa, | |
| Mucus of the gall-bladder, | 1.34 |
| | 1000.00 |

Of the above ingredients of the bile, we have the biliverdine, which is simply a coloring matter, the fats, with the oleates, margarates, and stearates, which, with the biliary salts, are said to hold the cholesterine in solution, the chloride of sodium, present in all the animal fluids, the phosphates and carbonates, which are simply excreted, and are also ingredients of the urine, leaving, as the most important constituents, of which the function is least understood, the biliary salts and the cholesterine. The biliary salts are probably recrementitious; but the cholesterine is one of the great products of the waste of the system. The bile, then, presents the combined character, so far as its chemical composition is concerned, of a secretion and of an excretion. Let us now contrast these two properties, and see what this fluid has in common with the secretions, and how it obeys the laws which regulate the excretions. In doing this we will first contrast some of the important distinctions between these two classes of products.

*Secretions* are characterized by certain elements which are manufactured in the substance of the gland, and are found in no other situation. Such is the pancreatine for the pancreatic juice, the pepsin for the gastrid juice, the ptyaline for the saliva, and, we may add, the glyco-cholate and tauro-cholate of soda for the bile.

These substances first make their appearance in the substance of the gland itself; they do not pre-exist in the blood; they are discharged from the gland for a special purpose, and when there is no necessity for their action, the discharge does not take place. Illustrations of this are to be found in the digestive fluids, which are true secretions; only poured when this function is called into action by the ingestion of food, and not discharged from the body, but their elements taken up again by the blood when their function is accomplished. Thus the gastric or pancreatic fluids are never secreted until food is taken into the alimentary canal, and are resorbed with the digested matters.

The flow of the secretions is intermittent, and the gland, during the period of repose, manufactures the elements of the secretion, which are washed out at the duct when the appropriate stimulus (of food, for example) causes a determination of blood to the organ. The gland manufactures the elements of the secretion, and the blood furnishes the menstruum, the water, by means of which they are dissolved and emptied into the duct. If we expose the pancreas of an animal during the intervals of digestion, it is pale and bloodless; no fluid flows from the duct; but the elements of the pancreatic juice are, nevertheless, in the gland, for if we macerate it in water, we may dissolve them out, and make an artificial pancreatic juice which will have all the reactions and digestive properties of the natural secretion. But if we expose the pancreas of an animal during digestion, the gland is turgid with blood; the secretion flows from the duct, and the products of the gland are being washed out by the blood—a process which we imitated when we dissolved them out by maceration in water. The late brilliant experiments of Bernard have shown that the function of the glands is regulated by the nervous system, and that the galvanization of certain nerves, by which the nervous force is imitated, will cause a determination of blood to the organ, and induce secretion, while the galvanization of other nerves will contract the vessels, and arrest secretion.

The substances which characterize the secretions, as they are manufactured in the glands and do not pre-exist in the blood, do not accumulate in the blood when the gland is removed, or its functions are interfered with.

The distinctive characters of the secretions, in fact, may be summed up thus:—

Their elements first appear in the glands, and do not pre-exist in the blood. They are not discharged from the body (with the exception of the milk, which is destined for the nourishment of the child). Their flow is intermittent. They are destined to assist in some of the nutritive functions of the body.

*Excretions*, of which the urine may be taken as a type, have entirely different characteristics.

Excrementitious substances do not first make their appearance in the organs which separate them, but are produced in the general system.

They pre-exist in the blood, having been absorbed by this fluid from the parts of the system in which they are formed, are carried to particular organs, and separated from the blood for the sole purpose of being expelled from the body. An illustration of this is to be found in the urea, which has been detected in the blood and urine, and some of the tissues of the body. This substance, one of the most important excrementitious products, is absorbed by the blood from certain parts of the system, carried to the kidneys, there separated from the blood, and discharged from the body. Though the gastric and pancreatic fluids, and all the secretions proper, are resorbed with the food after they have acted upon it, the urea may remain any length of time in the bladder, but it is never absorbed.

The flow of the excretions is constant. No period of repose is necessary for the gland to manufacture their elements, as they all pre-exist in the blood. Nutrition is constant, and destructive assimilation, or waste, which necessitates nutrition or repair, is likewise constant. The blood supplies all the wants of the system, and receives all the products of its decay. As the blood is continually being impoverished, it must be regenerated from without; and this is done by food, which is prepared for absorption by digestion. The secreted fluids are mostly concerned in digestion, and as this is an occasional process, the secretions are intermittent. But waste is continually going on, and excrementitious substances are continually forming; and while the necessity for the secretions is occasional, the necessity for the excretions is constant. Though the actual discharge of the latter from the body is occasional, they are constantly being separated from the blood, and accumulate in receptacles, whence they are discharged at appropriate intervals. No such receptacles exist for the secretions proper, except in the instance of the milk, which accumulates in the ducts of the mammary gland, and is the only secretion which is discharged from the body.

If the secreting glands take on an excretory function, as is an occasional pathological occurrence, their flow becomes continuous. We have an example of this in the occasional separation of the urea from the blood by the gastric tubuli. When the kidneys become so affected by disease as to be unable to separate the urea from the system, the accumulation of this excrement in the blood frequently induces other organs to attempt its removal. The gastric tubuli take on that function, and produce a fluid which contains urea. The gastric juice, if we may now so term it, is no longer a secretion, but an excretion, and we find that its flow is no longer intermittent and dependent upon the stimulus of food introduced into the stomach, but is constant, and continues until the irritation caused by the decomposing urea in the stomach induces an inflammation which prevents further secretion. Thus we have an example of an intermittent *secretion*, characterized by a substance manufactured in the gland and not pre-existing in the blood, changed into a constant *excretion*, characterized by a substance which is not manufactured in the gland but pre-exists in the blood.

The substances which characterize the excretions accumulate in the blood when the organ which eliminates them is removed, or its functions are interfered with. It is to this fact that we owed our knowledge that urea pre-existed in the blood. It was detected in that fluid when it had accumulated in animals from which the kidneys had been removed, and in cases of Bright's disease of the kidneys, before our chemical processes were sufficiently delicate to detect it in healthy blood, when the quantity is kept down to a very low standard by its constant elimination by the kidneys.

The characters of the excretions, then, are entirely opposite to those of the secretions.

Their elements pre-exist in the blood, and are not manufactured in the substance of the organs which eliminate them. Their flow is constant. They are separated from the blood merely to be discharged from the body, and are not destined to assist in any of the nutritive functions of the body.

Having thus contrasted the secretions and the excretions, let us examine the bile and note what are the characters which it has in common with either or both of these products.

The bile is characterized by two kinds of principles. One of them, the glyco-cholate and tauro-cholate of soda, *manufactured* in the liver, found in no other fluid than the bile, does not pre-exist in the blood, and associates the bile with the secretions. The other, the cholesterine, pre-exists in the blood and is simply *separated* from it by the liver, giving the bile one of the characters of an excretion.

The biliary salts (the glyco-cholate and tauro-cholate of soda) are discharged into the intestinal canal for a special purpose; and this discharge takes place at the beginning of the digestive act. If we expose the liver and gall-bladder of a dog which has not taken food, we will find the gall-bladder distended with bile; but if we examine these organs when digestion is going on, the gall-bladder will be found nearly empty. It is true that after prolonged fasting the bile is discharged into the alimentary canal, but it must be remembered that it contains another ingredient, the cholesterine, which must be discharged from the body, as we will see presently. The biliary salts are not discharged from the body. Dr. Dalton has shown that the substances extracted from the contents of the large intestine by evaporation, extraction of the residue with alcohol and precipitation with ether, will not react with Pettenkoffer's test, which is a very delicate test for the biliary salts. I have treated the feces of the human subject in the same way with the same result. These salts, therefore, are not discharged from the body unchanged. The next question to determine is whether they are discharged from the body in a modified form. They contain a certain amount of sulphur, of which, as has been shown by Bidder and Schmidt, only one-fifteenth part of the entire quantity which enters the intestine with the bile can be detected in the feces. As sulphur is an elementary substance, it cannot be decomposed; and the biliary salts, in this passage down the alimentary canal, must be absorbed. It is true that these salts cannot be detected in the blood coming from the intestines, but we cannot detect the pancreatin of the pancreatic juice, the pepsin or lactic acid of the gastric juice in the portal blood, yet these are absorbed by the mucous membrane of the intestinal tube, changed by their union with the elements they have digested. It is probable that an analogous change takes place in the glyco-cholate and taurocholate of soda, which prevents them from being detected in the blood by the ordinary tests. These facts, also, place the bile among the secretions.

On the other hand, cholesterine pre-exists in the blood, having been absorbed by this fluid from certain parts of the system, is carried to the liver, and here separated for the sole purpose of being discharged from the body. The same general remarks apply to this substance as to the urea. This places the bile among the excretions.

The flow of the secretions is intermittent. This is not absolutely true of the bile, but the discharge of this fluid is remittent. Dr. Dalton* has reported a series of interesting experiments upon an animal with a duodenal fistula. In this observation ten grains of dry biliary matter were discharged into the duodenum of a dog weighing thirty-six and a-half pounds, immediately after feeding. At the end of the first hour it had fallen to four grains; it continued at three and a-half to four and a-half grains up to the eighteenth hour, when the quantity was inappreciable; at the twenty-first hour it was one grain, the twenty-fourth, three and a-quarter grains, and the twenty-fifth three grains. The fluid was drawn for fifteen minutes each

* Dalton on the Constitution and Physiology of the Bile. Loc. cit.

time, evaporated to dryness, extracted with absolute alcohol, precipitated with ether, the ether precipitate dried, and weighed as representing the quantity of biliary matter present. These experiments apply to the time when the bile is discharged into the intestine; but as most animals have a gall-bladder, which collects the bile as it is secreted, it does not show when this fluid is formed by the liver. Schwann, Bidder, and Schmidt, Arnold, Kölliker, and Müller, have made experiments bearing upon the latter point, by ligating the ductus communis choledocus and making a fistula into the fundus of the gall-bladder. The experiments of these observers vary somewhat with regard to the time when the secretion of the bile is at its maximum. In the animal referred to on page 620, in which a fistula was made into the fundus of the gall-bladder, the bile was collected for thirty minutes immediately after feeding, one hour after, and then at intervals of two hours during the remainder of the twenty-four hours. The specimens of bile thus collected were carefully weighed, evaporated to dryness, and the proportion of dry residue taken. The accompanying table shows the results of these observations, which were made twelve days after the operation, when the animal, which weighed originally twelve pounds, had lost two pounds. His appetite was ravenous at the time of the experiment.

*Table of the variations of the bile in the twenty-four hours.* At each observation the bile was drawn for precisely thirty minutes. Dog with a fistula into the gall-bladder. Weight ten pounds.

| Time after feeding. | Fresh Bile. | Dried Bile. | Percentage of Dry Residue. |
|---|---|---|---|
| | grains. | grains. | |
| Immediately, . . . . | 8.103 | 0.370 | 4.566 |
| One hour, . . . . | 20.527 | 0.586 | 2.854 |
| Two hours, . . . . | 35.760 | 1.080 | 3.023 |
| Four hours, . . . . | 38.939 | 1.404 | 3.605 |
| Six hours, . . . . | 22.209 | 0.987 | 4.450 |
| Eight hours, . . . | 36.577 | 1.327 | 3.628 |
| Ten hours, . . . . | 24.447 | 0.833 | 3.407 |
| Twelve hours, . . . | 5.710 | 6.247 | 4.325 |
| Fourteen hours, . . . | 5.000 | 0.170 | 3.400 |
| Sixteen hours, . . . | 8.643 | 0.309 | 3.575 |
| Eighteen hours, . . . | 9.970 | 0.277 | 2.778 |
| Twenty hours, . . . | 4.769 | 0.170 | 3.565 |
| Twenty-two hours, . . | 7.578 | 0.293 | 3.866 |
| Twenty-four hours, . . | 15.001 | 0.885 | 5.233 |

This table shows a regular increase in the quantity of bile discharged from the fistula from the time of feeding up to four hours after. It diminished at the sixth hour, rose again at the eighth hour, but then gradually diminished to the fourteenth hour. We then have a slight increase at the sixteenth and eighteenth hours, and the twentieth hour it falls to its minimum. It then increased slightly the twenty-second hour, and mounted considerably the twenty-fourth hour, when the observations were concluded. Disregarding slight variations in the quantity, which might be accidental, it may be stated in general terms, that *the maximum flow of bile from the liver is from the second to the eighth hour after feeding; during which time it is about stationary. In this experiment it was at its minimum the twentieth hour after feeding.* This observation agrees with those of Bidder and Schmidt as regards the time when the bile begins to increase in quantity; but these observers state that it is at its maximum at the twelfth to the fifteenth hour. This, however, is not material to the question now under consideration. We wished to establish the fact that the quantity of bile secreted varied considerably during the various stages of the digestive act; a character which approximates it to other secretions. The flow of the bile is not intermittent, because it contains a substance which is excrementitious; but it is remittent, having a definite relation to the digestive act, because it contains substances which are recrementitious and are in some way connected with the process of digestion.

The continuous, though remittent, flow of the bile allies it with the excretions. There is no time, in health, when the bile is not separated from the blood. In animals that go through the process of hibernation, the bile continues to be secreted, though no food is taken into the alimentary canal. Nutrition, though much diminished in activity, goes on during this state, and the urea and cholesterine must be separated from the blood. The formation of the bile and urine, therefore, is not interrupted. The bile is secreted also in the fœtus, before any nourishment is taken into the alimentary canal, when none of the other digestive fluids are formed. This character it has in common with the urine, and this places it among the excretions.

The elements of secretion never accumulate in the system when the secretion is interfered with; while the elements of excretion do accumulate in the blood in such cases, and produce their toxic effects. Experimenters have often analyzed the blood for the biliary salts in cases of serious disease of the liver, marked by symptoms of bile poisoning, regarding these as the only important elements of the bile; but they have never been detected. I have made no observations on this point, for the fact that the glyco-cholates and tauro-cholates of soda do not accumulate in the blood in diseases of the liver has long been settled. This stamps these substances as products of *secretion;* but we will see when some of the pathological conditions of the cholesterine are taken up, that this substance does accumulate in the blood when the functions of the liver are seriously interfered with, which marks it as a product of *excretion.*

It seems to me that enough has been said with regard to the function of the bile to convince the reader that this complex fluid has two important elements which have two separate functions.

First. *It contains the glyco-cholate and tauro-cholate of soda; which are not found in the blood, are manufactured in the liver, are discharged mainly at a certain stage of the digestive process, are destined to assist in some of the nutritive processes, are not discharged from the body, and, in fine, are products of secretion.*

Second. *It contains cholesterine; which is found in the blood, is merely separated from it by the liver, and not manufactured in this organ, is not destined to assist in any of the nutritive processes, but merely separated to be discharged from the body, and is a product of excretion.*

These two propositions, and more especially the second, being established, it becomes our task now to follow out the cholesterine after it has been discharged from the liver into the small intestine. If it be discharged from the body it must be by the rectum, and to complete the history of cholesterine we find it necessary to study the feces.

*The Feces.*—It is not my object to consider all the effete matters which go to make up the feces, though it must be acknowledged that our information on this subject is very limited. Following the cholesterine in its passage down the alimentary canal has opened a new subject for investigation, which it will be impossible to do entire justice to in this paper. There is a field for a long series of investigations into this part of our subject, which I hope to be able to cultivate to some extent in the future, and add something to the history of the substance we have been considering. At present I shall only endeavor to demonstrate the fact that cholesterine, in a modified form, is discharged with the feces, and not attempt to treat of the conditions which modify the excretion of this substance (upon which as yet I have no data), which are of the last importance to the practical physician.

It is stated by some of the most reliable authors on physiology and physiological chemistry that cholesterine is found in the fecal matters. Robin and Verdeil say: "*Ce principe immediat se trouve a l' etat normal dans le sang, la bile, le foie, le cerveau, les nerfs, le crystallin et les matieres fecales.*" Many other authors refer to it as found in the feces, and it was with that belief, that, in the experiments which form the basis of this article, I deferred my analyses of the feces till I had completed the observations on the blood, and then analyzed them, satisfied that I would find cholesterine, with the view to determine the variations, etc., in its quantity. When after a careful and prolonged examination of many specimens of feces I was unable to extract any cholesterine, I endeavored to ascertain what observer had established its presence. Though it is mentioned by so many as present in the feces, I could find no mention of any one who had established this point; and in some of the analyses of Simon, I found that he had noted its absence in certain specimens of feces. I found also that Marcet, who published some elaborate analyses of the feces in the *Philosophical Transactions*, in 1854 and 1857, noted the absence of cholesterine in the normal feces of the human subject. We have already seen how conclusively the experiments on the blood from various parts of the system point to the excrementitious character of the cholesterine, showing us even in what part of the system it is found, and where it is eliminated; but it is undoubtedly one of the most important characters of an excretion that it should be discharged from the body, and I was unable for a time to convince myself that it was discharged. After evaporating the feces to dryness, pulverizing, extracting thoroughly with ether, decolorizing with animal charcoal, evaporating the ether and extracting the residue with boiling alcohol, I allowed the alcohol to evaporate, added a solution of caustic potash, and kept the mixture at a temperature near the boiling point for three and a-quarter hours. The potash was then carefully washed away in a filter, the residue redissolved in ether and extracted with hot alcohol as before, and the alcoholic extract set aside to evaporate. A number of days passed without any signs of crystallization. The residue was, of course, nonsaponifiable; but it differed from the cholesterine by being melted at a much lower temperature, though it presented the red color with sulphuric acid which is said to be characteristic of the latter substance. It was examined carefully with the microscope daily, and after five or six days, to my great satisfaction, crystals began to form; but they were at first so indistinct that their form could not be clearly made out. These crystals, however, increased in size and number, and in a short time presented all the characteristics of *seroline*. In about ten days the whole mass had crystallized, making one of the most superb exhibitions of crystals that could be imagined. The seroline crystallizes in the form of delicate transparent needles, which have a beauty under the microscope which could be but poorly imitated by the most delicate steel plate engraving. This substance, from its being found in such large quantities in the feces, I have spoken of as *stercorine*.

Before taking up the changes which the cholesterine undergoes in its passage down the alimentary canal, I will say a few words with regard to the stercorine, which will play an important part in this connection.

[*To be continued.*]

